# Development and Validation of Novel Nomograms Using Serum Tumor Markers for the Prediction of Preoperative Histologic Grades in Gastroenteropancreatic Neuroendocrine Tumors

**DOI:** 10.3389/fonc.2021.681149

**Published:** 2021-05-24

**Authors:** Yan Li, Zhi-Qi Wu, Qin Xu, Hemant Goyal, Hua-Guo Xu

**Affiliations:** ^1^ Department of Laboratory Medicine, The First Affiliated Hospital of Nanjing Medical University, Nanjing, China; ^2^ Department of Laboratory Medicine, Jurong Hospital Affiliated to Jiangsu University, Jurong, China; ^3^ Department of Internal Medicine, Mercer University School of Medicine, Macon, GA, United States

**Keywords:** GEP-NETs, Serum NSE, grade, nomograms, diagnosis

## Abstract

**Background:**

To develop and validate nomogram models for the preoperatively prediction of the histologic grade of gastroenteropancreatic neuroendocrine tumors (GEP-NETs) to provide appropriate treatments.

**Methods:**

A total of 1014 participants, including 211 healthy controls, 293 patients with benign diseases, 299 patients with cancers, and 211 patients with GEP-NETs were included in the final analysis. Their sociodemographic and laboratory information, including serum tumor markers such as AFP, CEA, CA19-9, CA72-4, Cyfra21-1 and NSE were collected. Nomogram models were developed to preoperatively predict histologic grades of GEP-NETs.

**Results:**

Among six serum tumor markers, only NSE was found to have a statistically significant association with the histologic grades in GEP-NETs (G1 *vs.* G2: p < 0.05; G2 *vs.* G3: p < 0.001; G1 *vs.* G3: p < 0.0001), which was combined with sex and age to develop the nomogram models. The first nomogram (to differentiate grade 1(G1) and grade 2/3 tumor (G2/G3)) showed a strong association to differentiate with an AUC of 0.747 (95% CI: 0.663-0.832) and 0.735 (95% CI: 0.624-0.847) in the training and validation datasets, respectively. The second nomogram (to differentiate G1/G2 and G3 tumors) showed a strong association to differentiate with an AUC of 0.827 (95% CI: 0.744-0.911) and 0.847 (95% CI: 0.744-0.950) in the training and validation datasets, respectively. The ROC, area under ROC curve (AUC), calibration curve and decision curve analysis (DCA) demonstrated the clinical usefulness of both models.

**Conclusions:**

We proposed two novel nomogram models based on sex, age and serum NSE levels to preoperatively predict the histologic grades in GEP-NETs to assist the clinical decision-making.

## Introduction

Neuroendocrine tumors (NETs) are heterogeneous malignancies arising from the diffuse neuroendocrine system. They can appear in various anatomic locations, but the majority of NETs are restricted to derivatives of the embryological gut, including the gastrointestinal (GI) tract and bronchopulmonary tree. Gastroenteropancreatic neuroendocrine tumors (GEP-NETs), including GI neuroendocrine tumors (GI-NETs) and pancreatic neuroendocrine tumors (pNETs) originate from enterochromaffin cells of the gutislets of Langerhans, respectively (1–5). Although GEP-NETs are rare, they comprise the second most common tumor of the digestive system after colorectal cancer. In the past few decades, the incidence rate of GEP-NETs has been increasing globally, which could be due to increased awareness and improvement in their detection methods (6–8).

According to the 2010 World Health Organization (WHO) grading system, well-differentiated NETs are classified as grade 1 (G1) and grade 2 (G2) tumors, and poorly-differentiated NETs are classified as the grade 3 (G3) tumors, based on the mitotic count and Ki-67 proliferation index (9). Tumor grade is a crucial determinant to guide the GEP-NETs management, but is usually determined on the postoperative specimens, which influencing the physician’s decision making in clinical practice (10). Recently, tissue acquision by EUS-guided fine needle aspiration (EUS-FNA) has helped evaluate the preoperative histologic grade, but with controversial accuracy (11, 12).

Various peptide hormones and biogenic amines secreted by GEP-NETs can enter systemic circulation, which could be used as biomarkers in the outpatient setting (13). At present, serum tumor markers are being extensively studied to provide future direction for the diagnosis, prediction and prognosis of the cancers (14). The neuron-specific enolase (NSE) is a cell-specific isozyme of the glycolytic enzyme enolase, which is highly specific for the neurons and peripheral nerve endothelial cells. Malignant neuron hyperplasia in the NETs may lead to an increase in the serum NSE level which can be used for the diagnosis, staging and treatment of these tumors, including GEP-NETs (15, 16). In this study, we have proposed two novel nomogram models to evaluate the role of NSE in the preoperative diagnosis and grade prediction of GEP-NETs.

## Materials and Methods

### Study Participants

A total of 1014 participants were included from the First Affiliated Hospital of Nanjing Medical University, China, between January 1, 2012 and December 31, 2019, including 211 healthy controls, 293 patients with benign diseases, 299 patients with cancers, and 211 patients with GEP-NETs were included in the final analysis. And the benign diseases includes gastroenteropancreatic inflammation and polyps. The diagnosis of various diseases was determined by practicing clinicians based on clinical guidelines. Exclusion criteria included: (a) patients with missing data; (b) patients with no histopathology; (c) patients who had already received treatment; (d) the samples showed hemolysis. Ethics committee approval was granted by the First Affiliated Hospital of Nanjing Medical University (Nanjing, China) ethics review board according to the Declaration of Helsinki. (Ethical approval No. 2020-SR- 012). Due to the retrospective nature of the study, informed consent was waived.

### Study Design

We collected the demographic information of the study participants, including sex, age and test results of six serum tumor markers, including alpha-fetoprotein (AFP), carcinoembryonic antigen (CEA), cancer antigen 19-9 (CA19-9), cancer antigen 72-4 (CA72-4), cytokeratin 19 fragment 21-1 (Cyfra21-1) and NSE. We compared the distribution of all these serum tumor markers in the study participants, including the HCs, benign disease, cancer and GEP-NETs groups. Next, in the GEP-NETs group, we compared the distribution and differences of serum NSE in different histologic grades as follows: Low grade or Grade 1 [G1] tumors have a mitotic rate of 0 to 1 per 10 high power fields (HPF) and a Ki-67 index of 0% to 2%, the mitotic rate of tumors of intermediate grade (G2) is 2 to 20 per 10 HPF or 3 to 20% of Ki-67 index and the mitotic rate of tumors of high grade (G3) is greater than 20 per 10 HPF or Ki-67 index 20% (9). According to the basic principles of variable selection in clinical prediction modelling, we selected the candidate variables for the model by the univariate logistic regression analysis and clinical knowledge (17). Comprehensively considering the significant levels in the two models (G1 *vs* G2/, G1/2 *vs* G3), variables with significant difference (p < 0.05) and clinical significance were chosen. Then, we chose the full model as the final model. The cutoff value of variable was decided according to the maximum Youden index of the ROC curve, which was used to turn into a binary variable.

### Statistical Analysis

Categorical variables are represented by frequency and proportion and continuous variables are represented by mean (standard deviation) and median (minimum and maximum). Because the distribution of the serum tumor markers in this study is right skewed, we transformed them to Normal distribution by taking log10, which made the prediction models more readable. The t-test or Mann–Whitney U test were used to evaluate the differences in the distribution of six tumor markers between the disease groups and the healthy control group. And NSE was compared in different grades of GEP-NETs.

Nomograms are based on the ratio of each regression coefficient to 0 to 100 points in the logarithmic regression conversion. The effect of the variable with the highest β coefficient (absolute value) is assigned 100 points. Add these points to the independent variables to get the total points and convert them into predicted probabilities. The predictive performance of the nomogram was measured by the area under the ROC curve (AUC) and the calibration curve with 1000 bootstrap samples. In addition, we performed the decision curve analysis, which calculates a clinical “net benefit” for the nomograms in comparison to default strategies of treating all or no patients. X-axis is preference, whose unit is High Threshold Probability. The Cost: Benefit Ratio help us see the relationship between preference and threshold probability easily. Y-axis shows the clinical decision net benefits after the benefits minus the disadvantages (18). R version 3.6.1 (http://www.rproject.org/) for all data analysis.

## Results

### Characteristics of Participants

Data was collected on a total of 1014 individuals during the study period. There were 211 healthy controls, 293 patients with benign diseases, 299 cancer patients, and 211 patients with GEP-NETs in the cohort with male patient accounting for 50.24%, 56.31%, 63.55% and 50.24%, respectively. Other demographic variables and information about the levels of six serum tumor biomarkers for these groups are shown in **Table 1**. The mean ages of the healthy controls, benign diseases, cancer patient and GEP-NETs patients were 54.43, 55.72, 62.7, and 54.36 years, respectively. The serum NSE levels were the highest in the patients with GEP-NETs. **Figure S1** showed the violin plots of six tumor markers in four groups. Three disease groups were compared with the healthy control group, respectively. Among all the tumor markers, NSE was significantly different in GEP-NETs (p < 0.0001) and had a smallest overlap with other disease groups. In addition, six tumor markers for distinguishing GEP-NETs from healthy and other disease groups were shown in the **Figures S2** and **S3**, respectively. Among them, serum NSE showed the best diagnostic performance.

**Table 1:** Characteristics of participants in four groups.

		HC	Benign	Cancer	GEP-NETs
		(n=211)	(n=293)	(n=299)	(n=211)
Sex					
	Male	106(50.24%)	165(56.31%)	190(63.55%)	106(50.24%)
	Female	105(49.76%)	128(43.69%)	109(36.45%)	105(49.76%)
Age, y					
	Mean (SD)	54.43(12.53)	55.72(15.58)	62.7(10.92)	54.36(12.64)
	Median [Min, Max]	55.00[20,81]	56.00[17,97]	64.00[30,84]	56.00[17,81]
AFP, ng/ml					
	Mean (SD)	3.50(2.03)	3.42(9.17)	7.25(61.81)	12.01(95.43)
	Median [Min, Max]	3.06[1.09,15.49]	2.43[0.6,154.90]	2.70[0.64,1056.00]	2.40[0.71,1210.00]
CEA, ng/ml					
	Mean (SD)	2.32(1.49)	2.26(2.69)	11.01(30.47)	8.53(69.20)
	Median [Min, Max]	2.05[0.37,13.28]	1.79[0.2,37.53]	3.15[0.62,340.8]	2[0.41,1000]
CA199, U/ml					
	Mean (SD)	13.61(7.96)	27.49(92.28)	135.22(255.15)	40.39(130.11)
	Median [Min, Max]	12.43[0.60,36.41]	11.12[0.60,1000.00]	24.38[0.60,1000.00]	10.86[0.60,1000.00]
CA724, U/ml					
	Mean (SD)	3.14(3.54)	3.39(17.70)	6.59(21.33)	4.52(20.97)
	Median [Min, Max]	2.00[0.31,30.40]	1.21[0.26,300.00]	2.02[0.30,300.00]	1.56[0.20,300.00]
Cyfra211, ng/ml					
	Mean (SD)	2.29(1.01)	1.7(0.95)	2.88(2.05)	2.77(5.38)
	Median [Min, Max]	2.03[0.79 6.30]	1.54[0.41,6.78]	2.46[0.67,23.67]	1.90[0.40,58.89]
NSE, ng/ml					
	Mean (SD)	12.22(1.82)	14.25(4.36)	16.17(5.98)	29.89(55.25)
	Median [Min, Max]	12.15[7.56,16.83]	13.76[4.64,31.14]	14.71[7.26,44.11]	16.14[8.57,467.50]

### Serum NSE in Different GEP-NETs Grades

**Figure 1:**
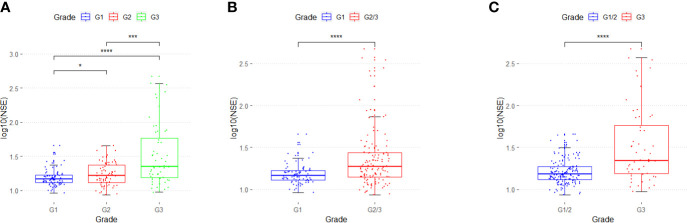
The distribution of serum NSE in different grades of GEP-NETs. **(A)** The serum NSE levels in G1, G2 and G3. **(B)** The serum NSE levels in G1 and G2/3. **(C)** The serum NSE levels in G1/2 and G3. (*p < 0.05; ***p < 0.01; ****p < 0.0001).

In 211 patients with GEP-NETs, the distribution of serum NSE level for different grades of GEP-NETs was significantly different (G1 *vs.* G2: p < 0.05; G2 *vs.* G3: p < 0.001; G1 *vs.* G3: p < 0.0001) (**Figure 1A**). In addition, NSE levels differ significantly between G1 and G2/3, and between G1/2 and G3 (p < 0.0001) grades of GEP-NETs (**Figures 1B, C** **)**.

### Nomogram to Differentiate G1 From G2/3 in GEP-NETs

**Figure 2:**
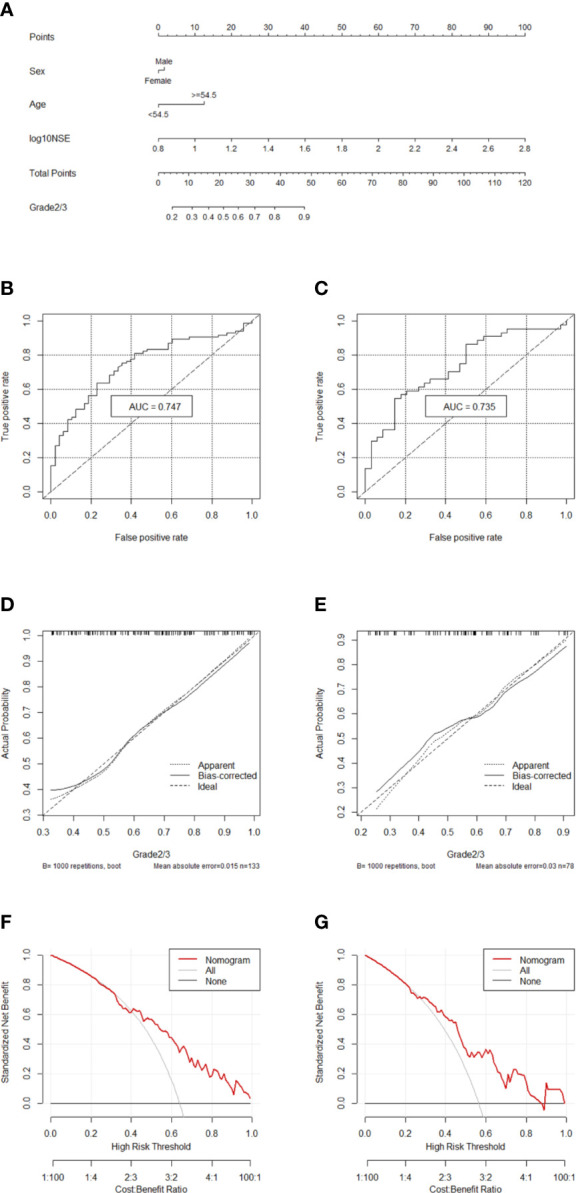
Nomogram for preoperatively predicting of G2/3 risk and its predictive performance. **(A)** Nomogram to estimate the risk of G2/3 preoperatively in patients with GEP-NETs. The area under the ROC curve [training datset: **(B)** validation dataset: **(C)**, the calibration curve (training datset: **(D)** validation dataset: **(E)**] and decision curve [trainingdata set: **(F)** validation dataset: **(G)**] of the nomogram.

Two hundred eleven patients with GEP-NETs were randomly divided according to the ratio of 7:3. There were 133 people in the training dataset, including 48 people in the G1 group and 85 people in the G2/3 group. Seventy eight individuals were included in the validation dataset, with 34 people in the G1 group and 44 people in the G2/3 group (**Table 2**). The results of the univariate logistic regression analysis showed that sex, age and NSE level was of clinical significance (**Table 1**). The nomogram to differentiate G1 from G2/3 in GEP-NETs was constructed based on the full model (**Figure 2A**). Age was used as a binary variable (<54.5 years and ≥54.5 years) according to the maximum Youden index of the ROC curve. NSE levels were transformed into the Normal distribution by taking log10. The AUC of the model reached 0.747 (95% CI: 0.663-0.832) and 0.735 (95% CI: 0.624-0.847) in the training and validation datasets, respectively (**Figures 2B, C** **)**. The calibration curve showed a high accuracy of the nomogram for predicting tumor pathologic grades both in the training and validation datasets (**Figures 2D, E** **)**. The DCA was used to demonstrate the clinical decision utility of the nomogram. The area under the decision curve in **Figures 2F, G** showed the clinical utility of corresponding strategies. The nomogram (red) showed more area than that the “treat all” (grey) or “treat none” (black) strategies, in both the training and validation datasets.

**Table 2:** Characteristics of patients with GEP-NETs in the G1 group and G2/3 group.

Characteristics	Training Dataset			Validation Dataset		
	(n=133)			(n=78)		
	Grade1	Grade2/3	p value	Grade1	Grade2/3	p value
	(n=48)	(n=85)		(n=34)	(n=44)	
Sex			0.348			0.198
Female	28 (58.33%)	41(48.24%)		19 (55.88%)	17 (38.64%)	
Male	20 (41.67%)	44(51.76%)		15 (44.12%)	27 (61.36%)	
Age, y			0.003			0.013
Mean (SD)	50. 27(11.43)	55.93 (12.48)		51.59 (13.20)	57.93 (12.54)	
Median [Min, Max]	50.50 [24, 81]	58.00 [21, 75]		52.00[25, 79]	61.50 [17, 75]	
AFP, ng/ml			0.046			0.484
Mean (SD)	2.55 (1.69)	25.64 (149.83)		2.78 (1.44)	3.12 (2.12)	
Median [Min, Max]	2.20 [0.71,10.90]	2.64 [0.88, 1210.00]		2.50 [1.10, 8.40]	2.70 [0.85, 13.16]	
CEA, ng/ml			0.195			0.122
Mean (SD)	2.07 (1.20)	15.80 (108.29)		2.15 (1.43)	7.88 (17.64)	
Median [Min, Max]	1.85 [0.60, 6.41]	2.00 [0.41, 1000.00]		1.74 [0.61,6.09]	2.24 [0.70, 89.42]	
CA199,U/ml			0.032			0.566
Mean (SD)	22.11 (62.17)	30.91 (91.18)		17.54 (23.78)	92.30 (240.04)	
Median [Min, Max]	7.81 [0.60, 424.2]	11.5[0.60,688.1]		11.66 [0.60, 135.7]	11.42 [0.600, 1000.00]	
CA724,U/ml			0.262			0.936
Mean (SD)	2.33 (2.72)	3.49 (5.39)		2.56 (2.66)	10.39 (45.07)	
Median [Min, Max]	1.45 [0.20, 14.45]	1.54 [0.45, 37.24]		1.67 [0.60, 11.86]	1.68 [0.25, 300.00]	
Cyfra21.1, ng/ml			0.049			0.386
Mean (SD)	2.38(2.60)	2.41 (1.34)		1.90 (0.75)	4.55 (11.22)	
Median [Min, Max]	1.70 [0.75, 15.90]	2.08 [0.51, 7.26]		1.66 [0.70, 3.90]	1.77 [0.40, 58.89]	
NSE, ng/ml			<0.001			0.079
Normal	15.27 (5.21)	32.80 (53.75)		16.98 (6.39)	52.15 (91.22)	
Abnormal	13.97[9.20, 35.92]	19.00 [8.57, 467.5]		15.21 [11.4, 45.49]	17.52 [9.20, 370.00]	

### Nomogram to Differentiate G1/2 From G3 in GEP-NETs

Two hundred eleven patients with GEP-NETs were still randomly divided according to the ratio of 7:3. There were 141 people in the training dataset, including 101 people in the G1/2 group and 40 people in the G3 group. Validation data included 70 people, including 54 and 16 people in G1/2 and G3 groups, respectively (**Table 3**).

**Table 3:** Characteristics of patients with GEP-NETs in the G1/2 group and G3 group.

Characteristics	Training Dataset			Validation Dataset		
	(n=141)			(n=70)		
	Grade1/2	Grade3	p value	Grade1/2	Grade3	p value
	(n=48)	(n=85)		(n=34)	(n=44)	
Sex			0.212			0.180
Female	58 (57.43%)	15(37.50%)		26 (48.15%)	6 (37.50%)	
Male	43 (42.57%)	25(62.50%)		28 (51.85%)	10 (62.50%)	
Age, y			<0.001			0.002
Mean (SD)	53.34(13.03)	62.08 (7.69)		48.89 (11.08)	60.00 (14.56)	
Median [Min, Max]	54.00 [21, 81]	62.00 [46, 75]		50.00 [23, 74]	65.00 [17, 75]	
AFP, ng/ml			0.318			0.575
Mean (SD)	9.94 (68.37)	32.97 (190.89)		2.84 (2.04)	3.61 (2.31)	
Median [Min, Max]	2.30 [0.71,689.7]	2.32 [0.85, 1210]		2.40 [0.75, 14.16]	2.95 [1.3, 9.8]	
CEA, ng/ml			0.071			0.002
Mean (SD)	2.41 (1.77)	34.93 (157.77)		1.87 (1.16)	3.74 (3.33)	
Median [Min, Max]	2 [0.41, 12.88]	2.45 [0.41, 1000]		1.46 [0.56,6.12]	2.41 [1.2, 12.06]	
CA199,U/ml			0.312			0.144
Mean (SD)	25.91 (65.15)	103.53 (255.2)		10.72 (8.38)	74.09 (150.71)	
Median [Min, Max]	11.83 [0.60, 492.30]	10.97[0.60, 1000.]		7.83 [0.60, 39.65]	18.69 [0.90, 470.30]	
CA724,U/ml			0.788			0.505
Mean (SD)	2.51 (2.64)	11.2 (47.12)		3.43 (5.41)	4.15(9.09)	
Median [Min, Max]	1.50 [0.20, 14.45]	1.80 [0.45, 300.00]		1.71 [0.20, 37.19]	1.08 [0.5, 37.24]	
Cyfra21.1, ng/ml			0.077			0.055
Mean (SD)	2.28(1.88)	4.08 (7.80)		1.86 (0.91)	5.65(14.24)	
Median [Min, Max]	1.88 [0.76, 15.9]	2.27 [0.51, 50.99]		1.75 [0.40, 5.06]	1.76 [0.72, 58.89]	
NSE, ng/ml			<0.001			0.003
Normal	17.67 (7.40)	65.18 (108.36)		16.75 (6.63)	63.99 (74.58)	
Abnormal	15.63[8.57, 45.49]	21.95 [9.40, 467.5]		14.96 [8.82, 38.00]	29.89 [10.6, 255.90]	

**Figure 3:**
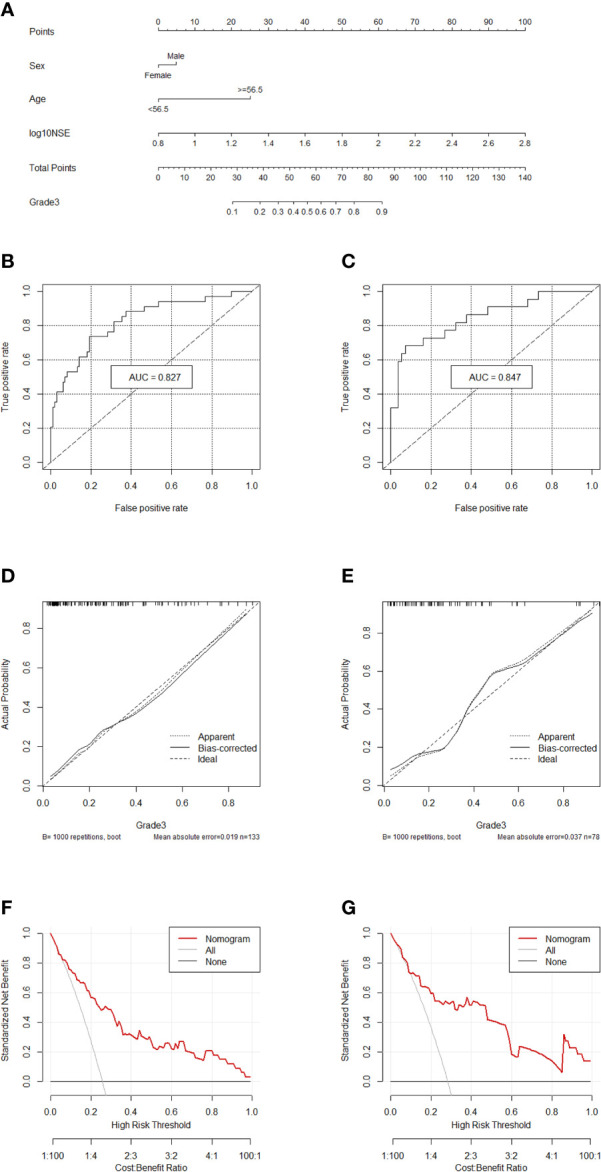
Nomogram for preoperatively predicting of G3 risk and its predictive performance. **(A)**, Nomogram to estimate the risk of G3 preoperatively in patients with GEP-NETs. The area under the ROC curve (training datset: **(B)**; validation dataset: **(C)**, the calibration curve (training datset: **(D)**; validation dataset: **(E)** and decision curve [trainingdata set: **(F)**; validation dataset: **(G)**] of the nomogram.

Comprehensively considering the results in the two models (G1 *vs* G2/, G1/2 *vs* G3), age, NSE and sex were chosen (**Table S1**). Then, we chose the full model as the final model and the nomogram to differentiate G1/2 from G3 in GEP-NETs was constructed (**Figure 3A**). Age was used as a binary variable (<56.5 years and ≥56.5 years) and NSE levels were taken log10. The AUC reached 0.827 (95% CI: 0.744-0.911) and 0.847 (95% CI: 0.744-0.950) in the training and validation datasets, respectively (**Figures 3B, C** **)**. The calibration curve showed a high accuracy of the nomogram for predicting tumor pathologic grades both datasets (**Figures 3D, E** **)**. The DCA was used to demonstrate the clinical decision utility of the nomogram. The area under the decision curve in **Figures 3F, G** showed the clinical utility of corresponding strategies.

## Discussion

In this study, we evaluated the role of serum NSE levels in the preoperative diagnosis and histologic grade prediction of GEP-NETs. Two novel nomogram models were established to predict the preoperative histologic grades to differentiate G1 and G2/3, and grades G1/2 and G3. The first model differentiated between G1 and G2/3 with an AUC of 0.747 (95% CI: 0.663-0.832) and 0.735 (95% CI: 0.624-0.847) in the training and validation datasets, respectively. The second nomogram differentiated between G1/2 and G3 with AUC of 0.827 (95% CI: 0.744-0.911) and 0.847 (95% CI: 0.744-0.950), respectively. The calibration curve and DCA demonstrated the clinical usefulness of these models.

NSE is localized in the neuronal and neuroendocrine cell cytoplasms and can be used as a circulating marker in GEP-NETs (19). However, NSE alone is not sufficient for the diagnosis of NETs, as only 30% to 50% of NETs secrete NSE (20). In a study involving more than 200 patients with GEP-NETs, the sensitivity and specificity of NSE to distinguish NETs from non-endocrine tumors were only 39-43% and 65-73%, respectively (13). However, an article showed that NSE has specificity for NETs than other tumor markers. In their study, all tumors positive for an accepted neuroendocrine marker also expressed NSE (21). In our study, AUC for NSE to distinguish GEP-NETs from healthy individuals was 0.819 (**Figures S2**). Nevertheless, in distinguishing GEP-NETs from other disease groups, the AUC was 0.657 (**Figures S3**). Despite these results, we believe that NSE still has a potential in the diagnosis of GEP-NETS among the six serological tumor markers.

In this study, serum NSE was found to be effective for GEP-NETs grade classification. GEP-NETs are heterogeneous in terms of origin, biological behavior with a malignant potential (5, 22). In the past few decades, various classification systems based on the embryological origin or morphological differences have been proposed for GEP-NETs (23, 24). The World Health Organisation (WHO) 2010 classified GEP-NETs in well-differentiated (G1 and G2) tumors, while poorly differentiated neuroendocrine carcinoma (NEC) are considered equivalent to G3 tumors (25). Different grades of GEP-NETs have different clinical severity and prognosis with different treatment approach (26, 27). In addition, different histologic grades have different prognosis based in the origin. In midgut GEP-NETs, the 5-year OS rates for G1, G2, and G3 tumors are 79%, 74%, and 40%, respectively (28). In pNETs, the 5-year OS of G1, G2, and G3 is 75%, 62%, and 7%, respectively (29). Therefore, it is crucial to predict the histologic grade of GEP-NETs preoperatively to help clinicians take decisive management actions effectively. A previous study proposed a combined nomogram model based on the radiomics signature and clinical-stage to distinguish G1 and G2/3 in pNETs for the treatment. In their study, parenchyma-sparing resections for G1 and a comprehensive treatment strategy including radical surgical resection with systematic chemotherapy was needed for patients with G2/3 to improve the survival (30). However, another study showed that the treatment strategies between G2 and G3 in pNETs should not be the same (31). These patients should receive surgical treatment in patients with limited metastatic disease, if technically feasible. Besides, targeted therapy with everolimus or sunitinib and somatostatin analogs (octreotide) is also used for advanced pNETs G1/2. Therefore, it is crucial to differentiate between G1/2 and G3 among pNETs than between G1 and G2/3 pNETs (31). Here, we developed two nomograms, one was used to distinguish G1 and G2/3 and the other was to distinguish G1/2 and G3.

A study showed that the average values of serum NSE for G1, G2, and G3 were 13, 17 and 21 μg/L in p-NETs, respectively (32). This was consistent with our results of NSE with significant differences in three grades of GEP-NETs. In our study, the nomogram differentiating G1/2 from G3 had a larger AUC than the nomogram differentiating G1 from G2/3. These results are consistent with the previous reports of advantages of NSE in diagnosing NETs with poor differentiation. In addition, elevation in NSE levels also reflected the overall survival of patients with GEP-NETs. Elevated serum NSE indicated the active disease, suggesting that the elevated NSE levels at the time of intial diagnosis is associated with poor prognosis (33). Therefore, the NSE can be used as a reliable diagnostic and prognostic markers in patients with GEP-NETs. We would also like to note some limitations of our study: (I) Relatively small sample size because it was a single-center study; (II) The information about the CgA was unavailable; (III) Inability to perform external validation of the data, and the conclusion in this study requires a larger multicenter validation analysis in future.

In summary, we reassessed the role of serum NSE in the diagnosis and prediction of preoperative histologic grades in GEP-NETs. We developed two novel nomogram models based on sex, age and serum NSE levels, which can be used as a non-invasive and accurate assessment tool for GEP-NETs patients during preoperative period to help clinicians tailor treatment plans accordingly.

## Data Availability Statement

The original contributions presented in the study are included in the article/**Supplementary Material**. Further inquiries can be directed to the corresponding author.

## Ethics Statement

The studies involving human participants were reviewed and approved by the First Affiliated Hospital of Nanjing Medical University (Nanjing, China) ethics review board. Written informed consent for participation was not required for this study in accordance with the national legislation and the institutional requirements.

## Author Contributions 

All authors contributed to the article and approved the submitted version.

## Funding

This work was supported by Natural Science Foundation of Jiangsu Province of China (BK20181492), the National Key Clinical Department of Laboratory Medicine of China in Nanjing, Key laboratory for Laboratory Medicine of Jiangsu Province (ZDXKB2016005) and by the Priority Academic Program Development of Jiangsu Higher Education Institutions. The protocol, data and analysis methods of this study are available on request. The sponsors and funders had no role in the study design, analysis of data or reporting.

## Conflict of Interest

The authors declare that the research was conducted in the absence of any commercial or financial relationships that could be construed as a potential conflict of interest.

## References

[1] Rindi G Wiedenmann B. Neuroendocrine Neoplasms of the Gut and Pancreas: New Insights. Nat Rev Endocrinol (2011) 8:54–64. 10.1038/nrendo.2011.120 21808296

[2] Cives M Strosberg JR. Gastroenteropancreatic Neuroendocrine Tumors. CA Cancer J Clin (2018) 68:471–87. 10.3322/caac.21493 30295930

[3] Modlin IM Oberg K Chung DC Jensen RT de Herder WW Thakker RV. Gastroenteropancreatic Neuroendocrine Tumours. Lancet Oncol (2008) 9:61–72. 10.1016/S1470-2045(07)70410-2 18177818

[4] Kunz PL. Carcinoid and Neuroendocrine Tumors: Building on Success. J Clin Oncol (2015) 33:1855–63. 10.1200/JCO.2014.60.2532 25918282

[5] Mafficini A Scarpa A. Genetics and Epigenetics of Gastroenteropancreatic Neuroendocrine Neoplasms. Endocr Rev (2019) 40:506–36. 10.1210/er.2018-00160 PMC6534496 30657883

[6] Lawrence B Gustafsson BI Chan A Svejda B Kidd M Modlin IM. The Epidemiology of Gastroenteropancreatic Neuroendocrine Tumors. Endocrinol Metab Clin North Am (2011) 40:1–18. 10.1016/j.ecl.2010.12.005 21349409

[7] Tian T Gao J Li N Li Y Lu M Li Z. Circulating Chromogranin A as A Marker for Monitoring Clinical Response in Advanced Gastroenteropancreatic Neuroendocrine Tumors. PloS One (2016) 11:e0154679. 10.1371/journal.pone.0154679 27159453 PMC4861261

[8] Dasari A Shen C Halperin D Zhao B Zhou S Xu Y. Trends in the Incidence, Prevalence, and Survival Outcomes in Patients With Neuroendocrine Tumors in the United States. JAMA Oncol (2017) 3:1335–42. 10.1001/jamaoncol.2017.0589 PMC5824320 28448665

[9] Kim JY Hong SM Ro JY. Recent Updates on Grading and Classification of Neuroendocrine Tumors. Ann Diagn Pathol (2017) 29:11–6. 10.1016/j.anndiagpath.2017.04.005 28807335

[10] Klimstra DS Modlin IR Coppola D Lloyd RV Suster S. The Pathologic Classification of Neuroendocrine Tumors A Review of Nomenclature, Grading, and Staging Systems. Pancreas (2010) 39(6):707–12. 10.1097/MPA.0b013e3181ec124e 20664470

[11] Di Leo M Poliani L Rahal D Auriemma F Anderloni A Ridolfi C. Pancreatic Neuroendocrine Tumours: The Role of Endoscopic Ultrasound Biopsy in Diagnosis and Grading Based on the WHO 2017 Classification. Dig Dis (2019) 37:325–33. 10.1159/000499172 30897588

[12] Vinayek R Capurso G Larghi A. Grading of EUS-FNA Cytologic Specimens From Patients With Pancreatic Neuroendocrine Neoplasms: it is Time Move to Tissue Core Biopsy? Gland Surg (2014) 3:222–5. 10.3978j.issn.2227-684X.2014.07.03. 10.3978/j.issn.2227-684X.2014.07.03 PMC4244506 25493252

[13] Hofland J Zandee WT de Herder WW. Role of Biomarker Tests for Diagnosis of Neuroendocrine Tumours. Nat Rev Endocrinol (2018) 14:656–69. 10.1038/s41574-018-0082-5 30158549

[14] Marotta V Zatelli MC Sciammarella C Ambrosio MR Bondanelli M Colao A. Chromogranin A as Circulating Marker for Diagnosis and Management of Neuroendocrine Neoplasms: More Flaws Than Fame. Endocr Relat Cancer (2018) 25:R11–29. 10.1530/ERC-17-0269 29066503

[15] Isgro MA Bottoni P Scatena R. Neuron-Specific Enolase as a Biomarker: Biochemical and Clinical Aspects. Adv Exp Med Biol (2015) 867:125–43. 10.1007/978-94-017-7215-0_9 26530364

[16] Kanakis G Kaltsas G. Biochemical Markers for Gastroenteropancreatic Neuroendocrine Tumours (GEP-Nets). Best Pract Res Clin Gastroenterol (2012) 26:791–802. 10.1016/j.bpg.2012.12.006 23582919

[17] Chowdhury MZI Turin TC. Variable Selection Strategies and its Importance in Clinical Prediction Modelling. Fam Med Community Health (2020) 8:e000262. 10.1136/fmch-2019-000262 32148735 PMC7032893

[18] Vickers AJ van Calster B Steyerberg EW. A Simple, Step-by-Step Guide to Interpreting Decision Curve Analysis. Diagn Progn Res (2019) 3:18. 10.1186/s41512-019-0064-7 31592444 PMC6777022

[19] Oberg K. Circulating Biomarkers in Gastroenteropancreatic Neuroendocrine Tumours. Endocr Relat Cancer (2011) 18(Suppl 1):S17–25. 10.1530/ERC-10-0280 22005113

[20] Sansone A Lauretta R Vottari S Chiefari A Barnabei A Romanelli F. Specific and Non-Specific Biomarkers in Neuroendocrine Gastroenteropancreatic Tumors. Cancers (Basel) (2019) 11. 10.3390/cancers11081113 PMC6721814 31382663

[21] Mjones P Sagatun L Nordrum IS Waldum HL. Neuron-Specific Enolase as an Immunohistochemical Marker Is Better Than its Reputation. J Histochem Cytochem (2017) 65:687–703. 10.1369/0022155417733676 28972818 PMC5714096

[22] Kim JY Hong SM. Recent Updates on Neuroendocrine Tumors From the Gastrointestinal and Pancreatobiliary Tracts. Arch Pathol Lab Med (2016) 140:437–48. 10.5858/arpa.2015-0314-RA 27128301

[23] WILLIAMS MS ED. The Classification of Carcinoid Tumours. Lancet (1963) 1:238–9. 10.1016/S0140-6736(63)90951-6 14000847

[24] Soga KT J. Pathologic Analysis of Carcinoids. Histologic Reevaluation of 62 Cases. Cancer (1971) 28:990–8. 10.1002/1097-0142(1971)28:4<990::AID-CNCR2820280424>3.0.CO;2-K 4106849

[25] Sorbye H Baudin E Perren A. The Problem of High-Grade Gastroenteropancreatic Neuroendocrine Neoplasms: Well-Differentiated Neuroendocrine Tumors, Neuroendocrine Carcinomas, and Beyond. Endocrinol Metab Clin North Am (2018) 47:683–98. 10.1016/j.ecl.2018.05.001 30098724

[26] Merola E Sbrozzi-Vanni A Panzuto F D'Ambra G Di Giulio E Pilozzi E. Type I Gastric Carcinoids: A Prospective Study on Endoscopic Management and Recurrence Rate. Neuroendocrinology (2012) 95:207–13. 10.1159/000329043 21811050

[27] Delle Fave G O’Toole D Sundin A Taal B Ferolla P Ramage JK. Enets Consensus Guidelines Update for Gastroduodenal Neuroendocrine Neoplasms. Neuroendocrinology (2016) 103:119–24. 10.1159/000443168 26784901

[28] Strosberg JR Weber JM Feldman M Coppola D Meredith K Kvols LK. Prognostic Validity of the American Joint Committee on Cancer Staging Classification for Midgut Neuroendocrine Tumors. J Clin Oncol (2013) 31:420–5. 10.1200/JCO.2012.44.5924 23248248

[29] Strosberg JR Cheema A Weber J Han G Coppola D Kvols LK. Prognostic Validity of a Novel American Joint Committee on Cancer Staging Classification for Pancreatic Neuroendocrine Tumors. J Clin Oncol (2011) 29:3044–9. 10.1200/JCO.2011.35.1817 21709192

[30] Liang W Yang P Huang R Xu L Wang J Liu W. A Combined Nomogram Model to Preoperatively Predict Histologic Grade in Pancreatic Neuroendocrine Tumors. Clin Cancer Res (2019) 25:584–94. 10.1158/1078-0432.CCR-18-1305 30397175

[31] Chen X Li B Wang S Yang B Zhu L Ma S. Efficacy and Safety of Endoscopic Submucosal Dissection for Gastrointestinal Neuroendocrine Tumors: A 10-Year Data Analysis of Northern China. Scand J Gastroenterol (2019) 54:384–9. 10.1080/00365521.2019.1588367 31037980

[32] Lv Y Han X Zhang C Fang Y Pu N Ji Y. Combined Test of Serum CgA and NSE Improved the Power of Prognosis Prediction of NF-Pnets. Endocr Connect (2018) 7:169–78. 10.1530/EC-17-0276 PMC5776672 29191920

[33] van Adrichem RC Kamp K Vandamme T Peeters M Feelders RA de Herder WW. Serum Neuron-Specific Enolase Level is an Independent Predictor of Overall Survival in Patients With Gastroenteropancreatic Neuroendocrine Tumors. Ann Oncol (2016) 27:746–7. 10.1093/annonc/mdv626 26712902

