# Supplementary material for: Development and Validation of Novel Nomograms Using Serum Tumor Markers for the Prediction of Preoperative Histologic Grades in Gastroenteropancreatic Neuroendocrine Tumors
Source: Front Oncol. 2021 May 24;11:681149. doi: 10.3389/fonc.2021.681149 (PMC8181758; doi:10.3389/fonc.2021.681149)
Supplement: Supplementary file 1 [file DataSheet_1.docx]

Supplementary Appendix
This appendix has been provided by the authors to give readers additional information about their work.
Supplement to: Development and validation of novel nomograms using serum tumor markers for the prediction of preoperative histologic grades in gastroenteropancreatic neuroendocrine tumors

**Development and validation of novel nomograms using serum tumor markers for the prediction of preoperative histologic grades in gastroenteropancreatic neuroendocrine tumors**

***Supplemental Materials***

**Table of Contents**

**eFigure 1. The violin plots of six tumor markers in four groups.**

**eFigure 2. The area under the ROC curve of distinguishing GEP-NETs from healthy group.**

**eFigure 3. The area under the ROC curve of distinguishing GEP-NETs from other disease groups**

**eTable 1. Univariate Logistic Regression Analysis in the Training Cohort**

**
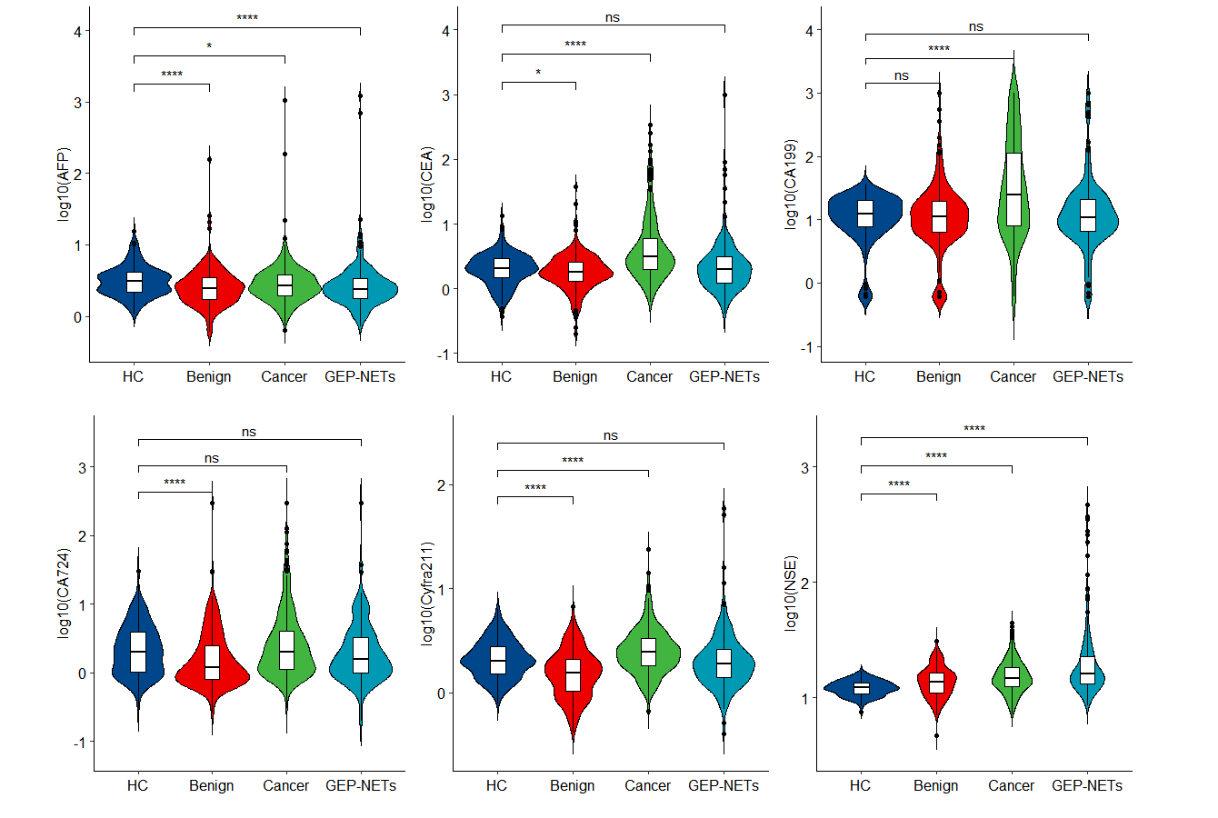
**

**eFigure 1. The violin plots of six tumor markers in four groups.**

**
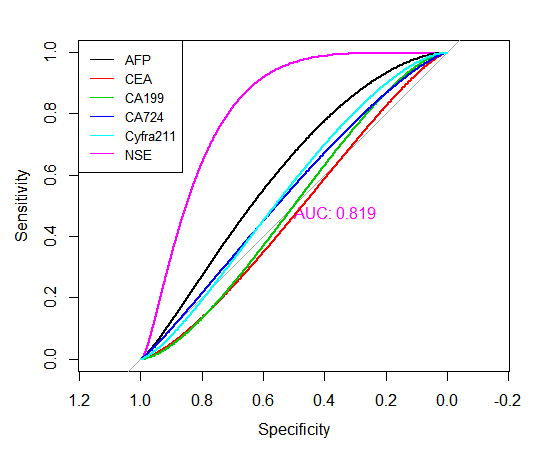
**

**eFigure 2. The area under the ROC curve of distinguishing GEP-NETs from healthy group.**

**
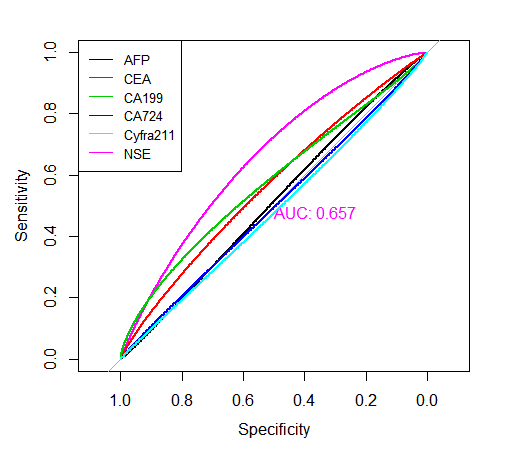
**

**eFigure 3. The area under the ROC curve of distinguishing GEP-NETs from other disease groups**

| **eTable 1. Univariate Logistic Regression Analysis in the Training Cohort** | | | | |
| --- | --- | --- | --- | --- |
| Characteristics | Grade1 vs Grade2/3 | | Grade1/2 vs Grade3 | |
|  | OR (95%CI) | P value | OR (95%CI) | P value |
| Sex(Female) | 0.67 (0.32 to 1.35) | 0.26 | 0.56 (0.25 to 1.23) | 0.15 |
| Age, y | 1.04 (1.01 to 1.07) | 0.013 | 1.09 (1.05 to 1.14) | <0.001 |
| AFP , ng / ml | 1.17 (1.00 to 1.49) | 0.119 | 1.00 (1.00 to 1.01) | 0.29 |
| CEA , ng / ml | 1.20 (1.01 to 1.58) | 0.136 | 1.24 (1.05 to 1.56) | 0.043 |
| CA199 ,U/ml | 1.00 (1.00 to 1.01) | 0.56 | 1.00 (1.00 to 1.01) | 0.283 |
| CA724 ,U/ml | 1.09 (0.98 to 1.26) | 0.19 | 1.09 (1.01 to 1.22) | 0.059 |
| Cyfra21.1 , ng / ml | 1.01 (0.84 to 1.25) | 0.92 | 1.13 (0.93 to 1.40) | 0.2 |
| NSE , ng / ml | 1.11 (1.05 to 1.19) | 0.001 | 1.07 (1.04 to 1.12) | <0.001 |
